# Acral Lentiginous Melanoma: A Case Control Study and Guidelines Update

**DOI:** 10.1155/2011/670581

**Published:** 2011-04-06

**Authors:** Christoforos Kosmidis, Christoforos Efthimiadis, Georgios Anthimidis, Marios Grigoriou, Kalliopi Vasiliadou, Georgia Ioannidou, Fotini Makedou, Sofia Baka

**Affiliations:** ^1^Department of Surgery, Interbalkan European Medical Center, 84 Tsimiski Street, 54622 Thessaloniki, Greece; ^2^Department of Radiology, “Panagia” General Hospital, 22 Nik. Plastira street, Nea Krini, Kalamaria, 55132 Thessaloniki, Greece; ^3^Department of Oncology, Interbalkan European Medical Center, 10 Asklipiou street, Pylaia, 57001 Thessaloniki, Greece

## Abstract

*Background*. Malignant melanoma incidence is increasing dramatically. We report herein a case of the rarest acral lentiginous type. *Case Report*. A 58-year-old man presented with a melanoma resembling lesion over the sole of his right foot, measuring 15–20 mm in diameter. An excisional biopsy with a narrow (2 mm) margin of surrounding skin was obtained. Histological findings were consistent with a diagnosis of acral lentiginous melanoma. Sentinel lymph node biopsy was also performed and micrometastases were not identified in frozen-section examination. According to the AJCC system, the tumor stage was IB (T2aN0M0). A wide local excision of the biopsy scar with a margin of 2 cm was performed. A split-thickness thick skin graft was used to reconstruct the excisional defect. During an 18-month followup, no local or distant recurrence has been observed. This paper aims to extract an updated rational approach to the management of this disease out of an enormous body of knowledge.

## 1. Introduction

The incidence of malignant melanoma is rising at a rate faster than any other form of cancer [1]. However, the mortality rate has fallen over the years, probably as the result of increased public awareness and education programs leading to earlier detection and treatment [2]. There are four distinct categories of melanoma. These are, in order of decreasing frequency, superficial spreading, nodular, lentigo maligna, and acral lentiginous. We report herein a case of the rarer acral lentiginous type and aim to extract a rational approach to the management of this disease out of an enormous body of knowledge.

## 2. Case Presentation

**Figure 1:**
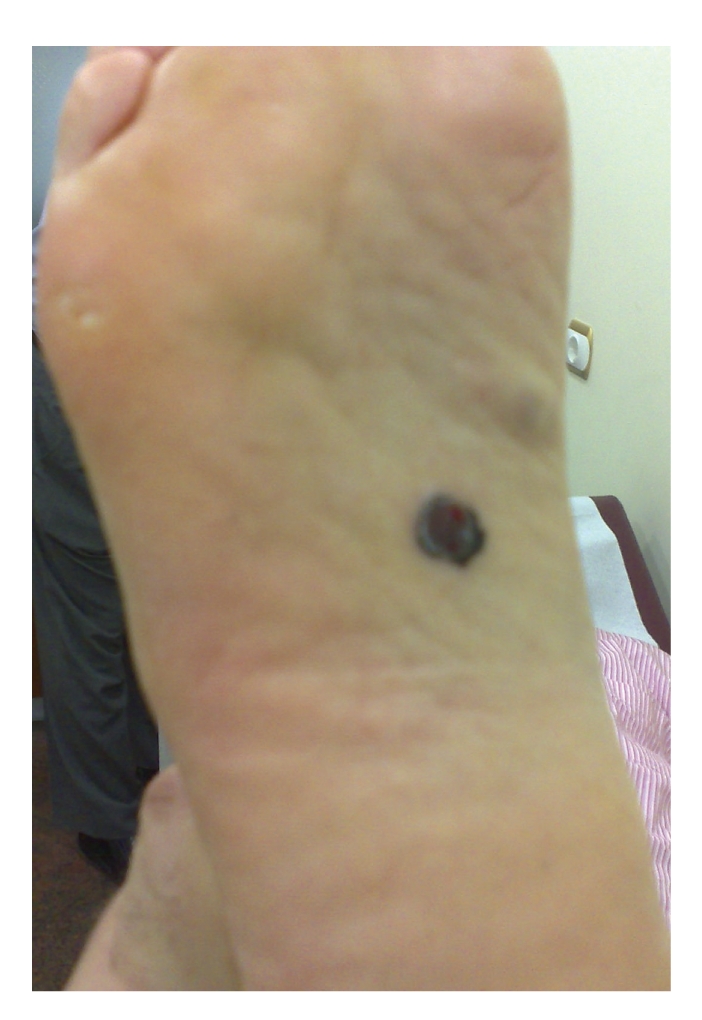
The lesion over the sole of patient's right foot, measuring 15–20 mm in diameter.

**Figure 2:**
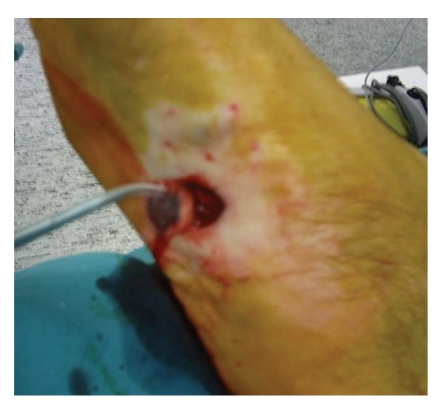
Perform of a wide local excision of the skin and underlying subcutaneous tissues with a margin of 2 cm.

**Figure 3:**
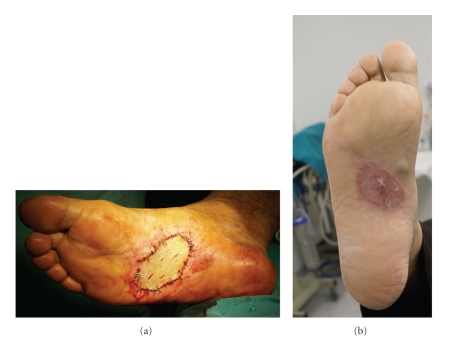
Use of a split-thickness thick (>0.016 inches) skin graft from anterior surface of the right thigh in order to reconstruct the excisional defect and provide an excellent aesthetic result.

A 58-year-old man presented with a 12-month history of a flat lesion that spread over the sole of his right foot and later became elevated, measuring 15–20 mm in diameter (Figure 1). Clinically, the lesion resembled acral lentiginous melanoma (ALM). Cutaneous examination was otherwise normal; no dysplastic or congenital nevi were identified. He was in otherwise good health, with no predisposing factors (family or personal history of melanoma, blond or red hair, freckling of the upper back, blistering sunburn before age 20, actinic keratosis, and blue, green, or gray eyes). There was no evidence of clinically apparent lymph node (inguinal) metastasis. The patient underwent an excisional biopsy of the suspicious lesion, including a layer of the underlying fatty tissue and the entire visible tumor, under local anesthesia. A narrow (2 mm) margin of surrounding skin was obtained and the wound was closed primarily in two layers in an orientation that was consistent with a possible subsequent wider excision. Histological findings were consistent with a diagnosis of ALM: intradermal features showing a diffuse proliferation of large atypical melanocytes along the epidermal-dermal junction which was dispersed in a lentiginous pattern with marked acanthosis and elongation of the rete ridges. The complete pathologic report included the following: Breslow thickness: 1,6 mm, absence of ulceration, Clark level II, negative surgical margins, absence of satellitosis, and absence of regression. Following the diagnosis, the patient was subjected to a thorough diagnostic evaluation to determine the possible spread of the disease to other sites. History focused on constitutional, central nervous system, pulmonary, gastrointestinal, and soft tissue symptoms; physical examination included a detailed inspection and palpation of the skin and subcutaneous tissue to detect satellites, in-transit metastases, other primary tumors, and lymph node enlargement; chest X-ray and abdominal ultrasonography were all negative for the presence of disease. Sentinel lymph node biopsy was consequently performed. The first (sentinel) lymph node draining the tumor was identified, removed, and since micrometastases were not identified in frozen-section examination, a complete lymph node dissection was not performed. According to the American Joint Committee on Cancer (AJCC) system of TNM (tumor, node, metastasis) classification, the tumor stage was IB (T2aN0M0). A wide local excision of the skin and underlying subcutaneous tissues was performed with a margin of 2 cm (Figure 2). A split-thickness thick (>0.016 inches) skin graft was used to reconstruct the excisional defect, providing an excellent aesthetic result (Figures 3(a) and 3(b)). The donor site was the anterior surface of the right thigh, which was left to heal by secondary intention. During an 18-month followup, no local or distant recurrence was observed.

## 3. Discussion

The term ALM was first described by Reed as a subtype of melanoma [3]. It was so named owing to its predilection of acral areas of the body, in particular the palms, soles, and the subungual areas, and its distinct radial or “lentiginous” growth phase. Although ALM is the rarest subtype of cutaneous melanoma, it represents the most common category diagnosed on the foot [4].

Clinical management of melanoma begins with an accurate diagnosis. Clinicians should have a low threshold to perform a diagnostic biopsy on any changing lesion. In our case the decision to perform a biopsy of the suspicious lesion was based on clinical experience.

A 1–3 mm margin of normal skin is taken if the wound can be closed primarily. Wider margins should be avoided to permit accurate subsequent lymphatic mapping. If removal of the entire lesion creates too large a defect, then punch biopsy or excision of a representative segment of the lesion is recommended. Once a diagnosis of melanoma is made, the biopsy scar and any remains of the lesion need to be removed to eradicate any remaining tumor. The size of the surgical margins depends on the tumor thickness. For in situ lesions a 0.5- to 1-cm margin of normal skin is adequate for cure. Thin melanomas (≤1 mm) require a 1-cm margin to prevent local recurrence; lesions between 1,01 and 2 mm should have a margin of 1-2 cm. For lesions between 2,01 and 4 mm, a 2 cm margin is recommended. Extending the resection beyond 2 cm does not appear to decrease local recurrence rates. Melanoma of fingers and toes requires digital amputation [5, 6]. 

The surrounding tissue should be removed down to the superficial fascia to remove all lymphatic channels. If the deep fascia is not involved by the tumor, removing it does not affect recurrence or survival rates, so the fascia is left intact. Generally, the wounds should be closed primarily. Larger tissue defects may be closed with local rotational/advancement skin flaps or a skin graft [7]. 

Evidence of tumor in regional lymph nodes is a poor prognostic sign. This is accounted for in the staging system by advancing any T classification from Stage I or II to Stage III. All clinically positive lymph nodes should be removed by regional nodal dissection unless unresectable distant metastases are present. Therapeutic lymph node dissection includes a superficial inguinal lymphadenectomy. The deep (iliac and obturator) nodes should be removed in the presence of clinical or radiographic evidence of deep node involvement or if there are more than three positive superficial nodes or when Cloquet's node is positive [8–11]. 

For patients with lesions less than 0.75 mm the tumor cells are still localized in the surrounding tissue, and treatment of regional lymph nodes is not beneficial. With lesions more than 4 mm, it is highly likely that the tumor cells have already spread to the regional lymph nodes and distant sites. Removal of the lymph nodes has no effect on survival [12, 13]. In patients with intermediate-thickness tumors (0.76 to 4.0 mm) and no clinical evidence of nodal or metastatic disease the efficacy of sentinel lymph node (SLN) biopsy has been established [13–18]. By using a combination of isotope lymphatic mapping, an intraoperative hand-held gamma probe, and intraoperative injection of blue dye, the SLN can be identified in more than 95% of cases in the groin and axilla. The sentinel node(s) is (are) removed, and if micrometastases are identified in frozen-section examination, a complete lymph node dissection is performed. Recently, detailed pathologic analysis of the sentinel nodes via step sections and immunohistochemistry along with diagnosis of submicroscopic disease based on genetic changes detectable by the polymerase chain reaction, enabled detection of micrometastases that could be missed by standard techniques [19].
